# 

**Published:** 1899-09

**Authors:** 


					CONTENTS.
The Antiseptic and Disinfectant
Properties of Soap.
Syuss, M.D. Lond., D.P.H.
... 193
On the Treatment of Subacute
Bronchitis.
By F. H. Edge-
worth, M B., B.Sc., B.A. Cantab.
Some Groups of Interesting Cases
of Abdominal Surgery.
By
Charles A. Morton, Jt\K.O.b.
Eng
203
Some Unusual Operations on Luna-
tics and their Results.
By J.
Paul Bush, M.R.C.S. Eng.
219
A Simple Form of Influence Machine
for X-Ray Work.
By William
Cotton, M.A., M.D. Ed.
... 222
Progress of the Medical Sciences:
Mcdiclne.
George Parker, M.D.
233
Surgery.
Thomas Carwardine ...
24$
Dermatology.
Henry Waldo, M.D.
249
Reviews of Books
254
Notes on Preparations for the Sick
280
The Library of the Bristol Medico-
Chirurgical Society 282
Local Medical Notes
287
Congres International de Medecine
29$
The College of Physicians of Phila-
delphia
294
Scraps
295

				

